# Identification of mitochondria-related biomarkers in childhood allergic asthma

**DOI:** 10.1186/s12920-024-01901-y

**Published:** 2024-05-23

**Authors:** Wei Zhao, Hongjuan Fang, Tao Wang, Chao Yao

**Affiliations:** Department of Pediatrics, The Second People’s Hospital of Hefei, Hefei, Anhui China

**Keywords:** Mitochondria-related genes, Childhood allergic asthma, GEO

## Abstract

**Background:**

The mechanism of mitochondria-related genes (MRGs) in childhood allergic asthma (CAS) was unclear. The aim of this study was to find new biomarkers related to MRGs in CAS.

**Methods:**

This research utilized two CAS-related datasets (GSE40888 and GSE40732) and extracted 40 MRGs from the MitoCarta3.0 Database. Initially, differential expression analysis was performed on CAS and control samples in the GSE40888 dataset to obtain the differentially expressed genes (DEGs). Differentially expressed MRGs (DE-MRGs) were obtained by overlapping the DEGs and MRGs. Protein protein interactions (PPI) network of DE-MRGs was created and the top 10 genes in the degree ranking of Maximal Clique Centrality (MCC) algorithm were defined as feature genes. Hub genes were obtained from the intersection genes from the Least absolute shrinkage and selection operator (LASSO) and EXtreme Gradient Boosting (XGBoost) algorithms. Additionally, the expression validation was conducted, functional enrichment analysis, immune infiltration analysis were finished, and transcription factors (TFs)-miRNA-mRNA regulatory network was constructed.

**Results:**

A total of 1505 DEGs were obtained from the GSE40888, and 44 DE-MRGs were obtained. A PPI network based on these 44 DE-MRGs was created and revealed strong interactions between ADCK5 and MFN1, BNIP3 and NBR1. Four hub genes (*NDUFAF7*, *MTIF3*, *MRPS26*, and *NDUFAF1*) were obtained by taking the intersection of genes from the LASSO and XGBoost algorithms based on 10 signature genes which obtained from PPI. In addition, hub genes-based alignment diagram showed good diagnostic performance. The results of Gene Set Enrichment Analysis (GSEA) suggested that hub genes were closely related to mismatch repair. The B cells naive cells were significantly expressed between CAS and control groups, and *MTIF3* was most strongly negatively correlated with B cells naive. In addition, the expression of *MTIF3* and *MRPS26* may have influenced the inflammatory response in CAS patients by affecting mitochondria-related functions. The quantitative real-time polymerase chain reaction (qRT‒PCR) results showed that four hub genes were all down-regulated in the CAS samples.

**Conclusion:**

*NDUFAF7*, *MTIF3*, *MRPS26*, and *NDUFAF1* were identified as an MRGs-related biomarkers in CAS, which provides some reference for further research on CAS.

**Supplementary Information:**

The online version contains supplementary material available at 10.1186/s12920-024-01901-y.

## Introduction

Asthma is a chronic airway disease characterized by eosinophilic inflammation of the airways, increased airway hyperresponsiveness (AHR), transformation of goblet cells, structural changes in the airways, elevated levels of Immunoglobulin E (IgE) levels, and reversible obstruction of airflow during exhalation [1]. Many environmental stimuli and genetic factors are known to contribute to the development of asthma [2, 3]. As a prevalent disease, asthma imposes a significant economic burden on society [4], patients, and their families. The diagnosis of childhood allergic asthma (CAS) primarily relies on patient’s medical history, clinical symptoms, and immunological examination, which has certain limitations [5] In recent decades, there has been a global increase in the incidence and mortality rate of asthma worldwide [6]. While currently available anti-asthma drugs are effective in controlling the clinical symptoms of asthma [7], they do not stop the natural progression of the disease. Therefore, it is necessary to carefully study the factors related to the progression of asthma in order to develop new therapeutic drugs for the treatment of CAS. About 50% of preschoolers experience wheezing at least once [8], but only a small number of children who wheeze go on to develop asthma. Currently, there is no “gold standard” for diagnosing CAS in children under the age of six. Therefore, the early detection of children who are at a high risk for asthma in CAS is critical. Notably, the early screening and effective prevention of high-risk CAS from a bioinformatics perspective will be of great significance for the occurrence and development of CAS. Given the extensive development of asthma research, asthma is now considered to be a complex and heterogeneous disease linked to mitochondrial genes [9, 10].

Mitochondrion (MT) is a well-known double-membrane organelle that is the power source for cells to produce energy in the form of ATP through oxidative phosphorylation (OXPHOS) in the electron transport chain [11]. Any change in the state within the mitochondria disrupts the OXPHOS mechanism, resulting in the accumulation and production of ROS [12], which in turn leads to mitochondrial dysfunction, forming a vicious cycle of cell damage and death [13]. Many studies [14, 15] have shown that MT play a crucial role in providing intermediates of oxidative metabolism through arginine/ornithine and may have an important influence on the signal transduction of airway inflammation. In addition, polymorphisms or haplotype differences in the mitochondrial genome have been found to be associated with asthma in humans [16]. The mitochondrial genome encodes the core catalytic peptides that form the main proteins in the electron transport chain [9]. Some studies [9, 17] have reported a link between allergic diseases and mitochondrial function. For example, MT provide intermediates for oxidative metabolism through arginine/ornithine Furthermore, they may have an important influence on signal transduction of airway inflammation. The pathophysiology of asthma is closely related to mitochondrial dysfunction [18], which leads to decreased ATP synthase activity, increased oxidative stress, apoptosis induction and abnormal calcium homeostasis [19–21]. Given the increasing incidence of CAS over the years, there is limited data on the role of mitochondria in mediating or regulating the effects of airway inflammation. No reports of a joint study between mitochondria-related genes (MRGs) and CAS have been found. Therefore, targeting mitochondrial dysfunction in the treatment of such diseases is currently promising and can be pursued as a research direction.

In this study, the public data of GEO database GSE40888 dataset was used to further screen the candidate characteristic genes for MRGs in CAS. We employed difference analysis, the lasso algorithm, and XGBoost algorithm. The genes obtained by the two algorithms were crossed to obtain four key genes: *NDUFAF7*, *MTIF3*, *MRPS26*, and *NDUFAF1*. We further explored four key genes, including expression correlation analysis and GSEA analysis, immune infiltration analysis, and inflammatory index analysis. In particular, the expression of *MTIF3* gene affected mitochondria-related functions, which negatively affected the immune system response to asthma, mainly in the abundance of B cell naive cells. It also positively affected the inflammatory response in asthma patients, suggesting that it may be able to reduce inflammation. It has influenced the onset and progression of CAS and could potentially be a novel approach to diagnosing and treating CAS.

## Materials and methods

### Data source

Two CAS-related datasets (GSE40888 and GSE40732) were obtained from the Gene Expression Omnibus (GEO) database (https://www.ncbi.nlm.nih.gov/gds). GSE40888 includes expression data from 41 blood samples of CAS and 86 blood samples of control samples. GSE40732 includes expression data from 97 blood samples of CAS and 97 blood samples of control samples. Additionally, 1136 MRGs were retrieved from the MitoCarta3.0 Database [22].

### Differential expression analysis

Differential expression analysis was performed between CAS and control samples using the limma R package [23] in the GSE40888 dataset to screen differentially expressed genes (DEGs) by setting |logFC| > 0.5 and *P.*adj < 0.05. And The Benjamini-Hochberg method was used to control for false discoveries generated when conducting multiple hypothesis tests. To better visualize the differences in gene expression among the two groups, the R packages ggpubr and ggplot2 [24] were used to plot volcano and heat maps of DEGs respectively. Differentially expressed MRGs (DE-MRGs) were obtained by overlapping the DEGs and MRGs. In addition, Gene ontology (GO) and Kyoto encyclopedia of genes and genomes (KEGG) enrichment analysis of DE-MRGs were completed using the clusterProfiler package [25] to explore the functions of it.

### Construction of protein protein interactions (PPI) network and acquisition of feature genes

To explore whether interactions existed among the DE-MRGs, a protein protein interactions (PPI) network was created using search tool for recurring instances of neighbouring genes (STRING, https://string-db.org). The CytoHubba plug-in in Cytoscape software was used to perform Maximal Clique Centrality (MCC) algorithms to obtain the degree ranking of each node in the above PPI network. The top 10 genes in the degree ranking were defined as feature genes.

### Acquisition of hub genes and construction of alignment diagram

To obtain valuable diagnostic genes, least absolute shrinkage and selection operator (LASSO) and EXtreme Gradient Boosting (XGBoost) based on feature genes were performed. The LASSO was completed by R package glmnet [26]. The XGBoost was completed by R package xgboost. The intersection of the diagnostic genes obtained by the two algorithms was defined as hub genes. Next, analysis of the expression trend of hub genes in CAS and control samples from GSE40888 and GSE40732 were finished. Moreover, to explore the potential mechanism of hub genes, Gene Set Enrichment Analysis (GSEA) for hub genes was conducted with ClusterProfiler package [25] in GSE40888.

Alignment diagram of hub genes were constructed in GSE40888 using the rms package [27] in R. The predictive power of the alignment diagram was assessed using calibration curves and decision curves. The closer the area under curve (AUC) of the receiver operating characteristic (ROC) curve was to 1, the more accurate of the diagnosis of the alignment diagram was. To further demonstrate the potential that hub genes can be used as biomarkers, we plotted the ROC curves of hub genes using pROC at GSE40888.

### Immuno-microenvironmental analysis and inflammatory microenvironmental analysis

The immune abundance of 22 immune cells in the CAS and control samples of the GSE40888 was calculated using the CIBERSORT algorithm [28]. After finding differentially expressed (DE)-immune cells between CAS and control samples, spearman correlations among the hub genes and between hub genes and DE-immune cells were calculated. A total of 200 inflammation-related genes (IRGs) were obtained from the HALLMARK_INFLAMMATORY_RESPONSE pass-through in the Molecular Signatures Database (MSigDB). The single sample gene set enrichment analysis (ssGSEA) of IRGs in all samples of the GSE127487 was conducted using the gsva [23] package in R to get the score of IRGs. Finally, the correlation between IRGs scores and hub genes was calculated.

### Construction of TF-miRNA-mRNA regulatory networks

To understand the regulatory role of hub genes in the pathogenesis of CAS, potential target miRNAs of hub genes were predicted by the miRNet database (https://www.mirnet.ca/miRNet/home.xhtml), the NetworkAnalyst database (https://www.networkanalyst.ca) was used to predict the transcription factors (TFs) that could interact with hub genes. Finally, based on the above miRNAs and TFs, the TF-miRNA-mRNA network was mapped using Cytoscape software.

### Quantitative real-time polymerase chain reaction (qRT‒PCR)

To further explore the role of hub genes in CAS, the expression of hub genes was validated by qRT-PCR. Totally 10 clinical blood samples in this experiment were obtained from normal and CAS patients in the Second People’s Hospital of Hefei including 5 normal and 5 CAS samples. Normal sample: Age range: 2–10 years old; no history of respiratory disease; no history of allergic disease; no infectious disease has occurred in the past 6 months.Sample of children with allergic asthma: Age range: 2–10 years old; the patient is clinically diagnosed with allergic asthma and meets one of the following criteria: (a) meets the World Health Organization (WHO) diagnostic criteria for asthma; (b) Have the characteristic clinical symptoms of asthma, such as wheezing, shortness of breath, chest tightness, etc. (c) Positive test results for allergen-specific IgE antibodies; (d) Response to bronchodilation test. Total RNA was prepared from blood samples using TRIZol reagent. Reverse transcription was performed using the SureScript-First-strand-cDNA-synthesis-kit to obtain cDNAs. The qRT-PCR was conducted as follows: totally 40 cycles, 95 °C for 1 min, 95 °C for 20 s, 55 °C for 20 s, and 72 °C for 30 s. GAPDH was used as the internal reference gene of hub genes. The qPCR primers were shown in Table 1. The relative expression levels of hub genes were calculated by 2^−ΔΔCt^ method.

**Table 1:** The information of primer sequences in quantitative real-time polymerase chain reaction (qRT-PCR)

Primer	Sequence
NDUFAF1 F	TCTTCTATAGGATTCACCTTGGCTG
NDUFAF1 R	AGCCTTGGGTTAAGCTCTGG
MTIF3 F	CAAAAGCCTTTAGTACCGCTGA
MTIF3 R	TGTTTCCCAAATCATTGCCCT
MRPS26 F	GCTGGCTATGTGCAGAGACTT
MRPS26 R	CCTCTTCCTCCGGGTCTTTG
NDUFAF7 F	TCCAGCCAAGCTACTAGGTAT
NDUFAF7 R	CGCTCTAACGGGACCTTCTC
GAPDH F	CGAAGGTGGAGTCAACGGATTT
GAPDH R	ATGGGTGGAATCATATTGGAAC

### Statistical analysis

Limma was used to identify DEGs. Venn was used for multiple gene sets to take intersections. LASSO and XGBoost were used to screen for hub genes. The ssGSEA for calculation of proportions of immune cells. GSEA was used to analyze the biological pathways in hub genes that may be involved. Statistical analysis was carried out through R software (https://www.r-project.org/). Differences between groups were analyzed via the Wilcox test. *P* < 0.05 represented a significant difference.

## Results

### DE-MRGs were associated with mitochondrial transcription

**Fig. 1:**
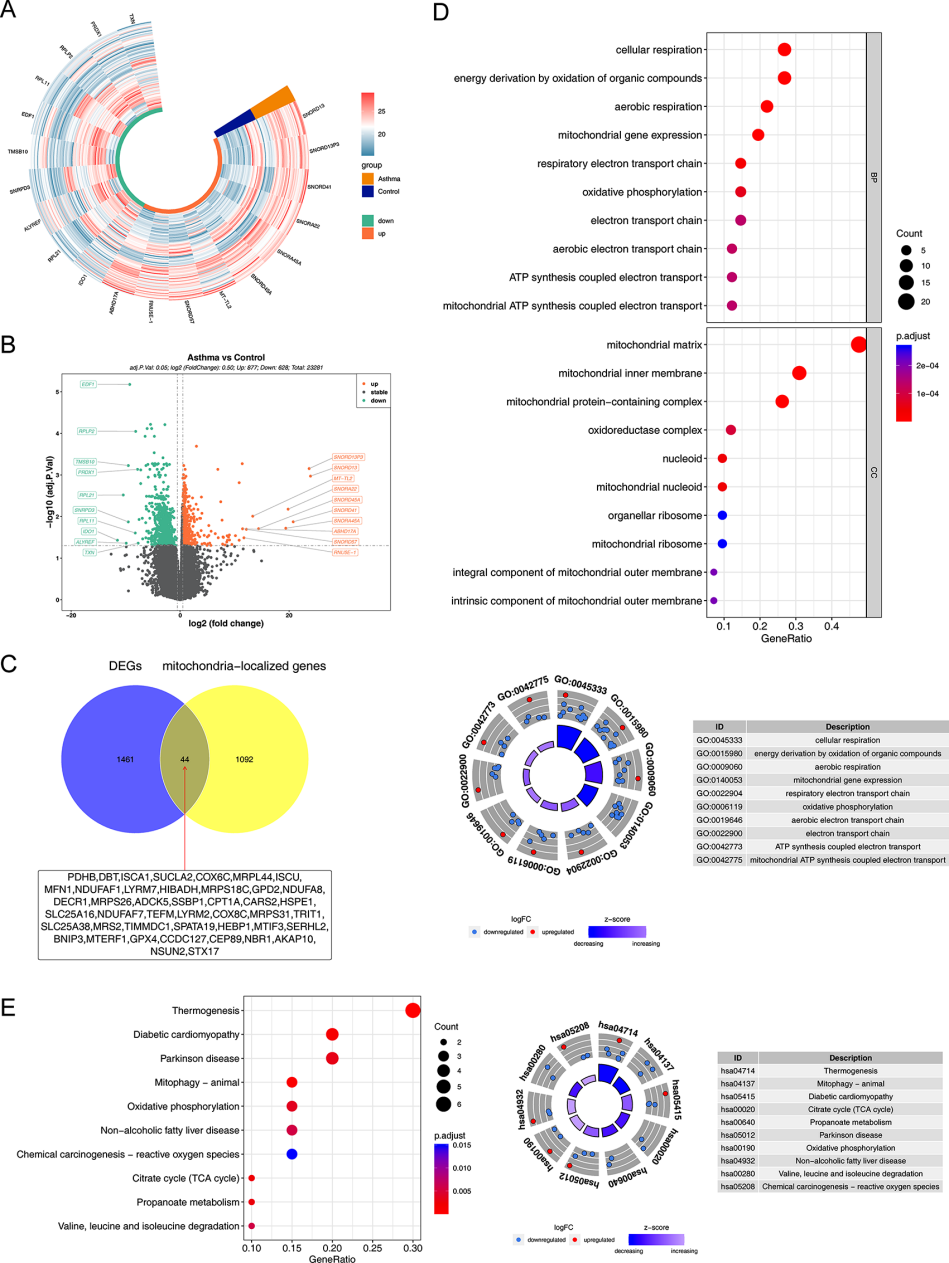
Identification of differentially expressed mitochondria-related genes (DE-MRGs). (**A**) The heatmap of differentially expressed genes (DEGs) from the GSE40888 in childhood allergic asthma (CAS). (**B**) The volcano plot of DEGs from the GSE40888 in CAS. (**C**) The Venn plot of DE-MRGs between DEGs and MRGs. (**D**) Gene ontology (GO) enrichment analysis of DE-MRGs (top: bubble char; bottom: circle chart). (**E**) Kyoto encyclopedia of genes and genomes (KEGG) enrichment analysis of DE-MRGs (left: bubble char; right: circle chart)

A total of 1505 DEGs were obtained from the GSE40888, and 877 DEGs were upregulated and 628 DEGs were downregulated in CAS (Fig. 1A-B). A total of 44 DE-MRGs were obtained by overlapping the 1505 DEGs and 1136 MRGs (Fig. 1C). Functional enrichment analysis revealed that DE-MRGs were associated with mitochondrial gene expression, mitochondrial RNA metabolic process, and mitochondrial transcription in GO entries (Fig. 1D), and enriched in propanoate metabolism, ferroptosis, and pyruvate metabolism in KEGG entries (Fig. 1E).

### Four genes (*NDUFAF7*, *MTIF3*, *MRPS26* and *NDUFAF1*) were identified as hub genes

**Fig. 2:**
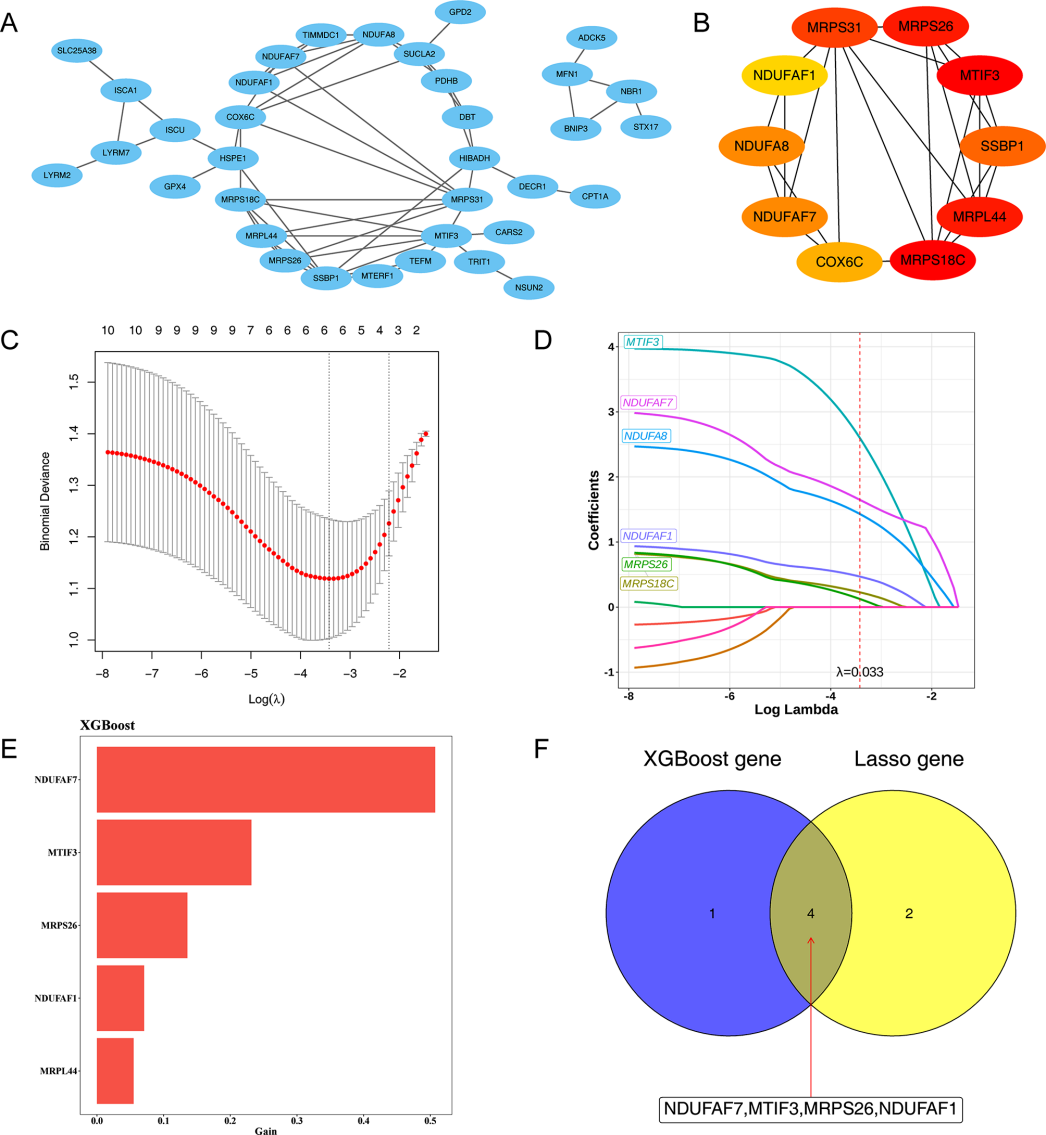
Screening of hub genes. (**A**) Construction of protein-protein interactions (PPI) network. (**B**) Top ten essential DE-MRGs ranked by maximal clique centrality (MCC) scores. (**C**) Least absolute shrinkage and selection operator (LASSO) regression analysis for screening the risk model genes. (**D**) The variation characteristics of the coefficient of variables. (**E**) Top five model genes selected using EXtreme Gradient Boosting (XGBoost) and the corresponding variable importance score. (**F**) The Venn plot of hub genes by taking the intersection of the XGBoost and LASSO algorithms

To explore whether interactions existed between the 44 DE-MRGs, a PPI network includes 35 nodes and 60 edges was created (Fig. 2A, Confidence = 0.4), there were strong interactions between ADCK5 and MFN1, BNIP3 and NBR1. The top 10 genes (*MTIF3*, *MRPS18C*, *MRPL44*, *MRPS26*, *MRPS31*, *SSBP1*, *NDUFAF7*, *NDUFA8*, *COX6C*, and *NDUFAF1*) in the degree ranking of MCC algorithms were defined as feature genes (Fig. 2B). The results of the LASSO regression analysis suggested that when λ = 0.033, six model genes with regression coefficients that are not penalized to 0 are obtained after tenfold cross-validation (Fig. 2C-D). Five model genes were obtained from the XGBoost model, and histograms of gene coefficients were plotted with larger Gini coefficients indicating the greater importance of the gene (Fig. 2E). Four hub genes (*NDUFAF7*, *MTIF3*, *MRPS26*, and *NDUFAF1*) were obtained by taking the intersection of the model genes of the above two models (Fig. 2F).

### Hub genes were closely related to pyrimidine metabolism

**Fig. 3:**
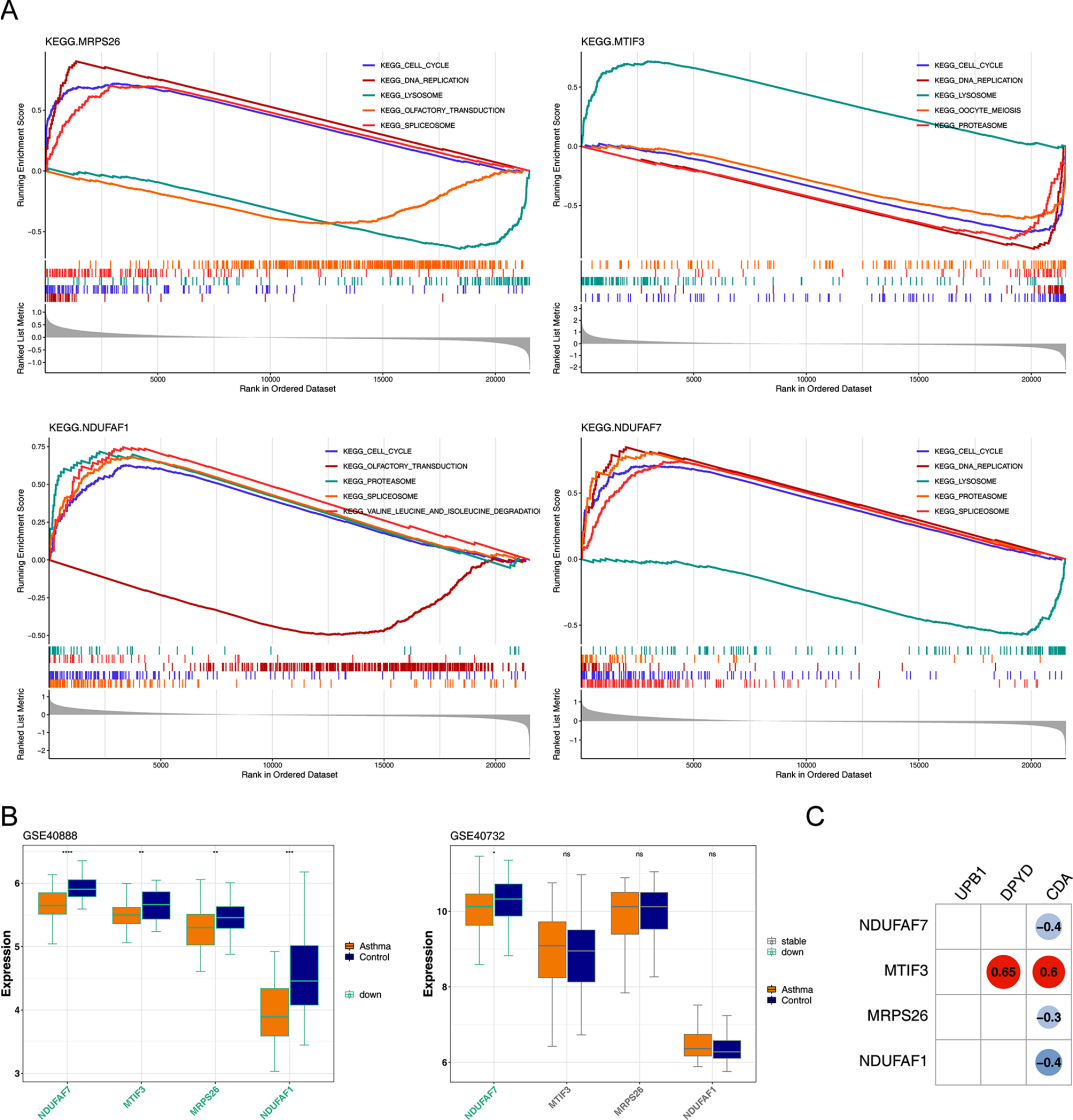
Functionality of four hub genes. (**A**) Gene set enrichment analysis (GSEA) of four hub genes. (**B**) The box plot of expression of four hub genes between CAS and control groups in GSE40888 dataset and GSE40732 dataset. (**C**) Correlation analysis of hub genes with pyrimidine metabolism, red represents positive correlation and blue represents negative correlation. * *p* < 0.05, ** *p* < 0.01, *** *p* < 0.001, **** *p* < 0.0001, ns, no significance

The results of GSEA suggested that the *MRPS26* was enriched in pyrimidine metabolism, mismatch repair, and homologous recombination in KEGG terms. *MTIF3* was enriched in mismatch repair, butanoate metabolism, and lysosome in KEGG terms. *NDUFAF7* was enriched in glycolysis gluconeogenesis, pyrimidine metabolism, and mismatch repair in KEGG terms. *NDUFAF1* was enriched in spliceosome, mismatch repair, and pyruvate metabolism in KEGG terms (Fig. 3A). In addition, the expression validation results of the hub genes suggested that *NDUFAF7* was down-regulated in CAS samples and showed consistent trends in the GSE40888 dataset and GSE40732 dataset, with other genes to be verified in further experiments. (Fig. 3B, *P* < 0.05). To further explore the correlation between hub genes and pyrimidine metabolism, we calculated the correlation between hub genes and β-Ureidopropionase (UPB1), Dihydropyrimidine Dehydrogenase (DPYD), and Cytidine Deaminase (CDA), and the results showed that *MTIF3* had a strong positive correlation with DPYD (Fig. 3C).

### Hub gene-based alignment diagram showed good diagnostic performance

**Fig. 4:**
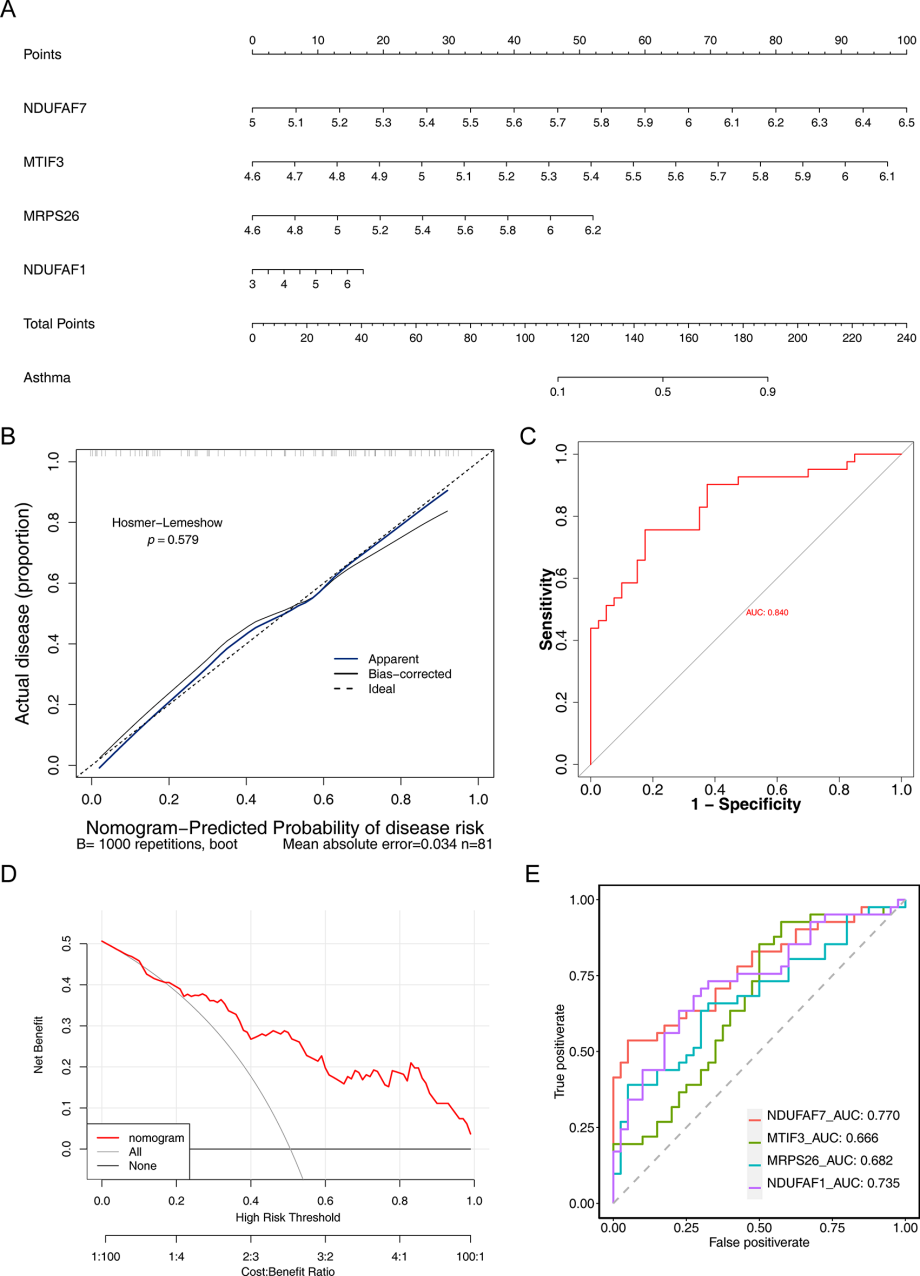
A nomogram prediction of hub genes in CAS samples. (**A**) Hub genes nomogram. (**B**–**D**) Calibration curves (left). Receiver operating characteristic (ROC) curves (middle) and decision curve analysis (DCA) (right) for the diagnostic nomogram. (**E**) ROC analysis of hub genes in GSE40888 dataset

Based on the results of the logistic regression model, an alignment diagram was constructed, and the score of each sample was calculated by the alignment diagram, the higher the total score of the patient, the higher the likelihood that the patient will develop CAS disease (Fig. 4A). The calibration curve and decision curve suggested that the ROC of the model was 0.840, indicating that the alignment diagram possesses good diagnostic efficacy (Fig. 4B-D). By plotting the ROC curves, we found that the AUC of hub genes in the GSE40888 dataset were all greater than 0.6, indicating that hub genes have the potential to be used as CAS biomarkers (Fig. 4E).

### *MTIF3* was strongly negatively correlated with B cells naive

**Fig. 5:**
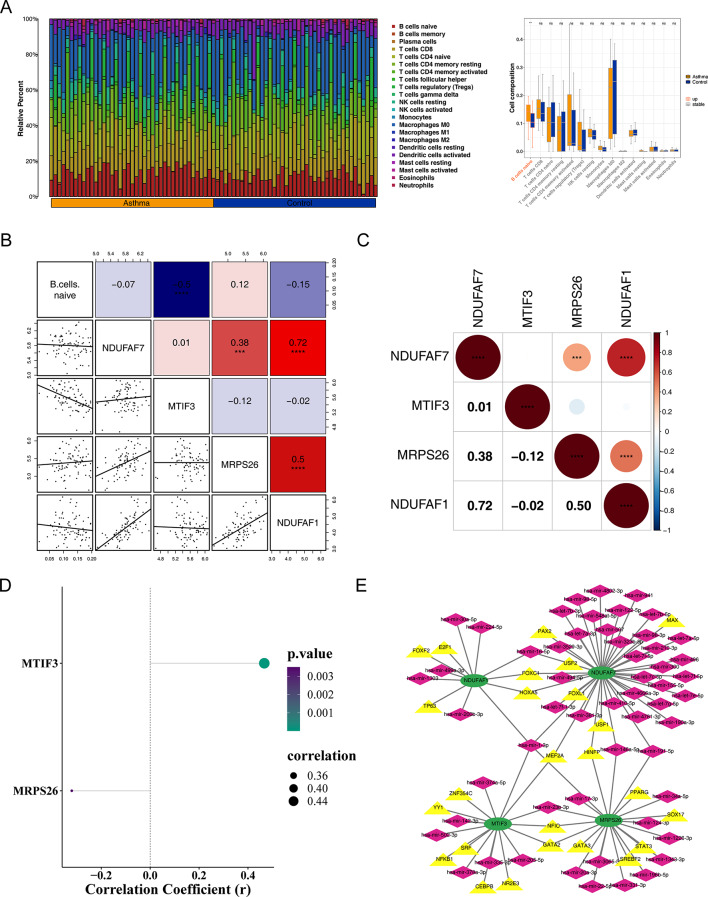
Immune correlation analysis. (**A**) Box plot for the expression levels of different cell types and the heat maps of the proportion of different immune cells in CAS and control groups. (**B**) The correlation analysis between hub genes and DE-immune cells. (**C**) The correlation analysis among hub genes. (**D**) The correlation analysis between hub genes and the score of inflammation-related genes (IRGs). (**E**) Construction of transcription factors (TFs) -miRNA-mRNA network. Green indicates hub genes, pink indicates miRNAs, yellow indicates TFs. * *p* < 0.05, ** *p* < 0.01, *** *p* < 0.001, **** *p* < 0.0001, ns, no significance

Immune cells with a cell abundance of 0 in more than 75% of the samples were filtered out, leaving 15 immune cells, and significant differences in the expression of B cells naive were observed between CAS and control groups (Fig. 5A). The results of the correlation analysis between hub genes and DE-immune cells suggested that *MTIF3* is the most strongly negatively correlated with B cells naive (Fig. 5B, Cor=-0.5, *P* < 0.0001). The results of the correlation analysis among hub genes suggested significant correlations between *NDUFAF7* and *MRPS26* (Cor = 0.38, *P* < 0.001), *NDUFAF7* and *NDUFAF1* (Cor = 0.72, *P* < 0.001), and *MRPS26* and *NDUFAF1* (Cor = 0.50, *P* < 0.0001) (Fig. 5C). The results of the correlation analysis between Hub genes and the score of IRGs suggested that *MTIF3* correlated significantly positively with IRGs score (Cor = 0.47) and *MRPS26* correlated significantly negatively with IRGs score (Cor=-0.32) (Fig. 5D). A total of 25 TFs and 55 miRNAs that may interact with hub genes were obtained, and a TF-miRNA-mRNA network was constructed (Fig. 5E). There were some relationships existed such as *NDUFAF1* acting on hsa-mir-499a-3p by influencing E2F1.

### Hub genes were confirmed by qRT-PCR

**Fig. 6:**
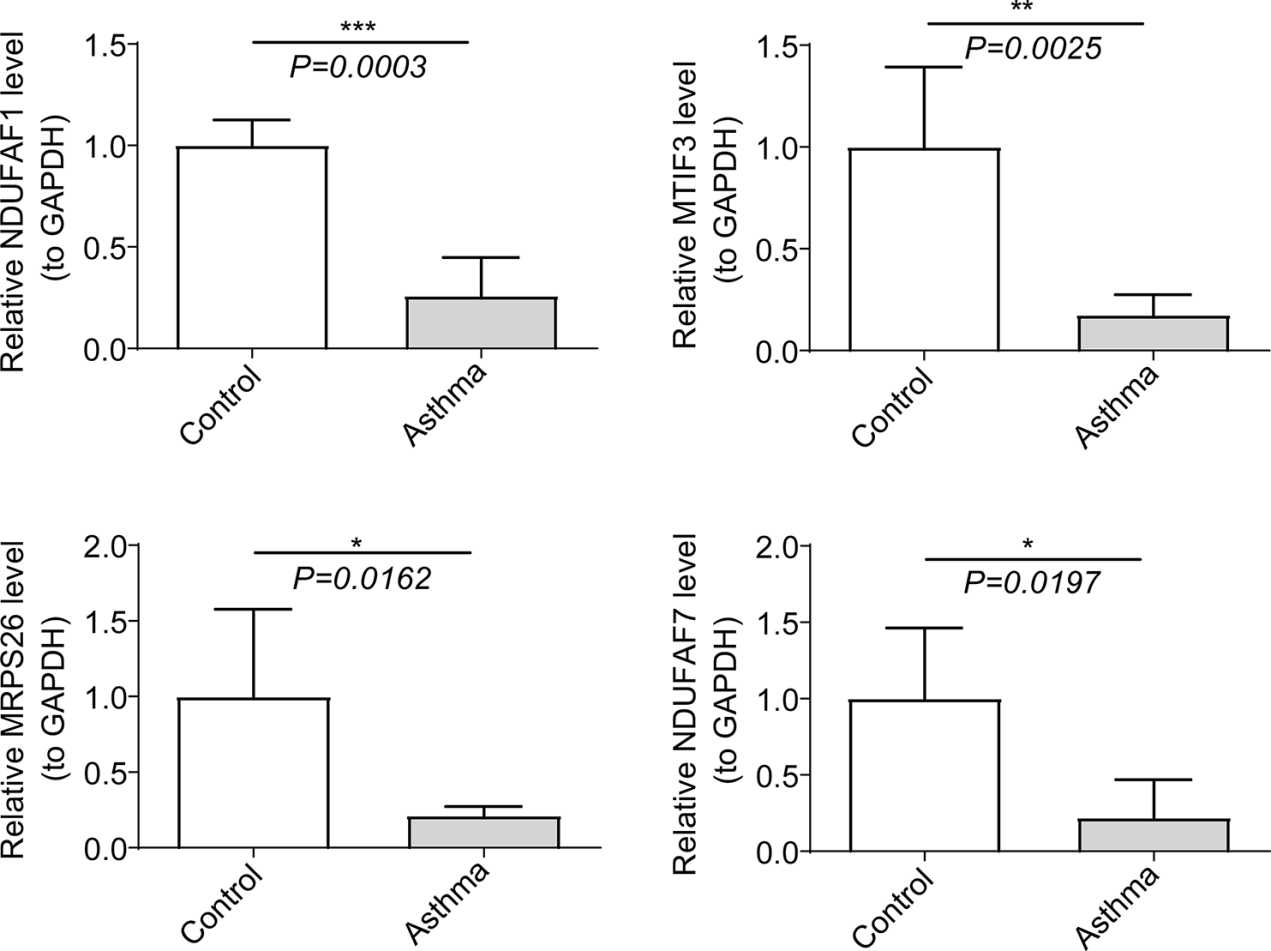
Validation of four hub genes (*NDUFAF7*, *MTIF3*, *MRPS26*, and *NDUFAF1*) expression by quantitative real-time polymerase chain reaction (qRT-PCR) in control and CAS blood samples. * *p* < 0.05, ** *p* < 0.01, *** *p* < 0.001

The qRT-PCR results showed that the expression levels of four genes (*NDUFAF1*, *MTIF3*, *MRPS26* and *NDUFAF7*) in the CAS group were significantly lower compared with the normal group (Fig. 6).

## Discussion

At present, there have been few studies on asthma and mitochondria-related genes. Therefore, the aim of our research is of great significance to as we explore mitochondrial related biomarkers in CAS. In this study, we investigated the hub genes (*NDUFAF7*, *MTIF3*, *MRPS26*, *NDUFAF1*) that can be used for accurate diagnosis of CAS. Significantly, we detected the expression of each hub gene and obtained the corresponding score. The incidence of CAS could be determined based on the total score.

Over-expression of the target protein amyloid precursor protein (APP) gene caused by exon variants of *NDUFAF7* is associated with the risk of Alzheimer’s disease [29]. Mammalian mitochondrial ribosomal protein *MRPS26* expression levels are correlated with tumor purity in non-small cell lung cancer (NSCLC). *MRPS26* has been observed to participate in mitochondrial activity of muscle stem cells [30]. *MTIF3* encodes 29 kDa proteins that promote the formation of initiation complexes on mitochondrial 55 S ribosomes and play an active role in translation initiation. The *MTIF3* gene may influence Parkinson’s disease by causing mitochondrial dysfunction [31]. In our study, in the GSE40888 dataset, the expression of *MTIF3* in the CAS sample was lower than that in the normal sample. This suggested that, similar to Parkinson’s disease, MTIF3 may trigger CAS by causing mitochondrial dysfunction. In addition, we found that *MTIF3* was negatively correlated with infantile B cells, suggesting that there could be an imbalance in the immune system. This negative correlation may lead to an abnormal response to the immune system, affecting the immune response and autoimmune response. Especially in CAS, it may lead to an abnormal response to the sensitivities. The negative correlation between *MTIF3* and the immature B cells may further aggravate the abnormal immune response, increase the inflammatory and bronchial contraction induced by the system, and promote the development of allergic asthma in children. Previous studies have shown that cell B cells play a key role in allergic diseases [32], while the decline of *MTIF3* levels may affect the function and differentiation of juvenile b cells, further exacerbating the immune disorder caused by the process. A Study [33] has demonstrated that *NDUFAF1* is an crucial mitochondrial protein involved in complex I assembly and stabilization, with important functions in electron transport and proton pumping. Expression levels of *NDUFAF1* in two central nodes involved in epidermal growth factor (EGF) and Olfactomedin 4 (OLFM4) have been associated with acute kidney injury and septic shock [34]. It is suggested that *NDUFAF1* is involved in the cellular inflammatory response pathway.

Some scholars have found that [14] MT function also has a positive effect on the inflammatory response of asthma patients, suggesting that inflammatory response may affect the occurrence and progression of asthma. Wu et al. [35] found that an iron chelator could relieve allergic airway inflammation. CAMKK2 and CISD1 are key suppressors of iron-induced cell death in asthma. CAMKK2 is down-regulated in patients with asthma. Interestingly, the negative correlation between CISD1 gene expression and Tregs suggests a potential role of CISD1 in regulating immune cell infiltration [36]. Tregs are viable targets of airway allergic inflammatory responses and play an integral role in maintaining immune tolerance in asthma [37]. By up regulating immunosuppressive molecules and suppressor genes, the Tregs portion of CD4 T cells prevents the development of pro-inflammatory activity and reduces inflammation [38, 39]. In our study, an analysis to examine the correlation between the hub gene and immune cells. The results revealed that the immune system’s response to asthma is negatively affected, mainly due to the high the abundance of B-cell naive cells. *MTIF3* had the strongest negative correlation with B cell naive. The activation of B cells in the airway during the inflammatory response is quite different from the classical B cell activation observed in secondary lymphoid organs, and has been demonstrated [40]. In addition, the expression of the *MTIF3* and *MRPS26* genes negatively affects the immune system’s response to asthma, impacting mitochondrial-related functions.

Lin et al. [41] showed that CAMKK2 participated in mitochondrial autophagy and promoted iron death. Autophagy may play a key role in chronic airway inflammation [42]. Increased autophagy plays an important role in airway remodeling, extracellular matrix deposition and fibrosis in asthma [43]. In addition, genetic mutations in autophagy genes were associated with asthma [44]. Particulate matter 2.5 (PM2.5) has been reported [45] to drive mitochondrial autophagy and induce cell cycle arrest and senescence. Our KEGG enrichment results indicate that differentially expressed mitochondria-related genes are associated with thermogenesis, mitochondrial phagocytosis - animal, diabetic cardiomyopathy, citric acid cycle (TCA cycle), propionic acid metabolism, Parkinson’s disease, oxidative phosphorylation, nonalcoholic fatty liver, valine, leucine, and isoleucine degradation. Additionally, these genes are correlated with chemo-9 - Carcinogenic effects - reactive oxygen species and other pathways. GSEA enrichment analysis based on KEGG gene set showed that the enrichment results of four hub genes were enriched in a total of one cell cycle pathway. Four hub genes regulate the occurrence of autophagy through some mitochondrial signaling pathways, resulting in cell cycle arrest and cell senescence, which contribute to the development of asthma phenotype. This is in line with some studies suggesting that cell senescence in the lung may be an important risk factor for asthma [46, 47]. And mitochondrial dysfunction has been shown to drive premature cellular aging, consistent with its impact on airway diseases [48, 49].

According to our research, we found a group of 25 TFs and 55 miRNA that interact with the hub gene, and built a TF-miRNA-mRNA network. In this network, we found that NDUFAF1 was used to affect E2F1 in the case of hsa-mir-499a-3p. These findings reveal the complex interactions between TFs and miRNA in regulating the network, providing important clues to the pathogenesis of CAS. These TFs and miRNA may have an impact on the development of CAS by regulating the hub genes and its relevant signaling pathways, affecting the process of inflammatory regulation, airway reconstruction and immune response. In addition, we also found that the hsa-mir-1-3p played a major role in the joint control hub gene. It is reported that the hsa-mir-1-3p has been found to be involved in the control of multiple diseases, such as hepatocellular carcinoma, alzheimer’s disease and type 2 diabetes, etc [50–52]. However, it is not clear how the specific mechanism in CAS is unclear, and further research will help clarify the exact role of these regulatory factors in the mechanism of asthma pathogenesis and provide theoretical basis for future development of more effective treatment strategies. At the same time, the TF-miRNA-mRNA network we established provides potential biomarkers and therapeutic targets for molecular diagnosis and targeted therapy for related diseases. In general, our study provides a new perspective and thought for the pathogenesis of CAS, and lays the foundation for future clinical transformation and personalized treatment.

This study is similar to previous research protocols, but is different in that it is a biomarker related to mitochondrial function in CAS, simple and straightforward. The results of this study were obtained from a dataset of blood samples. The chronic inflammation, vasoconstriction, and hyperresponsiveness of CAS often lead to systemic inflammatory changes. Therefore, it is possible to collect blood samples of CAS. Screening out dysregulated mitochondria could be a promising way to prevent or stop the development of these chronic lung diseases. The clinical application of the results of bioinformatics analysis requires more sample data support and further clinical experiments. It also shows that we will continue to pay attention to the role of these genes. However, this study has some limitations, especially the small sample size, which may impact the reliability and statistical significance of the results. Furthermore, due to the limitations of the sample type in the dataset, this study was unable to accurately determine the specific cell type associated with the observed differential expression. Nevertheless, this study offers a novel reference for CAS treatment.

## Conclusion

In this work, the study identified four biomarkers (*NDUFAF7*, *MTIF3*, *MRPS26*, and *NDUFAF1)* related to mitochondrial function in CAS using bioinformatics analysis and conducted correlation analysis to explore the mechanism of action, which provides some reference for further research on CAS.

### Electronic supplementary material

Below is the link to the electronic supplementary material.

Supplementary Material 1

## Data Availability

These databases, namely the GEO database (https://www.ncbi.nlm.nih.gov/gds) [GSE40888 and GSE40732], MiRNet database (https://www.mirnet.ca/miRNet/home.xhtml), and NetworkAnalyst database (https://www.networkanalyst.ca), are utilized in current research for data analysis.

## Abbreviations

MRGs: Mitochondria-Related Genes
CAS: Childhood Allergic Asthma
DEGs: Differentially Expressed Genes
DE-MRGs: Differentially Expressed Mrgs
PPI: Protein-Protein Interactions
MCC: Maximal Clique Centrality
LASSO: Least Absolute Shrinkage And Selection Operator
XGBoost: EXtreme Gradient Boosting
TFs: Transcription Factors
GSEA: Gene Set Enrichment Analysis
AHR: Airway Hyperresponsiveness
IgE: Immunoglobulin E
MT: Mitochondrion
OXPHOS: Oxidative Phosphorylation
ROS: Reactive Oxygen Species
GEO: Gene Expression Omnibus
GO: Gene Ontology
KEGG: Kyoto Encyclopedia of Genes And Genomes
AUC: Area Under Curve
ROC: Receiver Operating Characteristic
MSigDB: Molecular Signatures Database
IRGs: Inflammation-Related Genes
ssGSEA: Single Sample Gene Set Enrichment Analysis
APP: Amyloid Precursor Protein
NSCLC: Non-Small Cell Lung Cancer
EGF: Epidermal Growth Factor
OLFM4: Olfactomedin 4
PM2.5: Particulate Matter 2.5
TCA cycle: Tricarboxylic Acid Cycle

## References

[1] Busse WW, Lemanske RF (2001). Jr Asthma N Engl J Med.

[2] Mukherjee AB, Zhang Z (2011). Allergic asthma: influence of genetic and environmental factors. J Biol Chem.

[3] Ranjbar M, Whetstone CE, Omer H, Power L, Cusack RP, Gauvreau GM. The genetic factors of the Airway Epithelium Associated with the Pathology of Asthma. Genes (Basel). 2022;13. 10.3390/genes13101870 PMC9601469 36292755

[4] Vellopoulou K, Bakakos P, Loukides S, Maniadakis N, Kourlaba G (2019). The Economic Burden of Asthma in Greece: a cross-sectional study. Appl Health Econ Health Policy.

[5] Khan DA (2014). Allergic rhinitis and asthma: epidemiology and common pathophysiology. Allergy Asthma Proc.

[6] Keller MB, Lowenstein SR (2002). Epidemiology of asthma. Semin Respir Crit Care Med.

[7] Papi A, Brightling C, Pedersen SE, Reddel (2018). HK Asthma Lancet.

[8] Dai B, Sun F, Cai X, Li C, Liu H, Shang Y (2021). Significance of RNA N6-Methyladenosine regulators in the diagnosis and subtype classification of Childhood Asthma using the gene expression Omnibus Database. Front Genet.

[9] Jang H, Kim M, Hong JY, Cho HJ, Kim CH, Kim YH (2020). Mitochondrial and nuclear mitochondrial variants in allergic diseases. Allergy Asthma Immunol Res.

[10] Wang CM, Zhang XJ, Ma YJ, Li X (2017). Mutational analysis of mitochondrial tRNA genes in patients with asthma. Iran J Public Health.

[11] Reddy PH (2006). Amyloid precursor protein-mediated free radicals and oxidative damage: implications for the development and progression of Alzheimer’s disease. J Neurochem.

[12] Mailloux RJ (2016). Application of Mitochondria-Targeted Pharmaceuticals for the treatment of Heart Disease. Curr Pharm Des.

[13] Wang CH, Wu SB, Wu YT, Wei YH (2013). Oxidative stress response elicited by mitochondrial dysfunction: implication in the pathophysiology of aging. Exp Biol Med (Maywood).

[14] Qian L, Mehrabi Nasab E, Athari SM, Athari SS (2022). Mitochondria signaling pathways in allergic asthma. J Investig Med.

[15] Kim YH, Lee SH (2018). TGF-β/SMAD4 mediated UCP2 downregulation contributes to aspergillus protease-induced inflammation in primary bronchial epithelial cells. Redox Biol.

[16] Cocco MP, White E, Xiao S, Hu D, Mak A, Sleiman P (2020). Asthma and its relationship to mitochondrial copy number: results from the Asthma Translational Genomics Collaborative (ATGC) of the Trans-Omics for Precision Medicine (TOPMed) program. PLoS ONE.

[17] Iyer D, Mishra N, Agrawal A (2017). Mitochondrial function in allergic disease. Curr Allergy Asthma Rep.

[18] Thomas B, Rutman A, Hirst RA, Haldar P, Wardlaw AJ, Bankart J et al. Ciliary dysfunction and ultrastructural abnormalities are features of severe asthma. J Allergy Clin Immunol. 2010;126:722-9.e2. 10.1016/j.jaci.2010.05.046 20673980

[19] Ranjbarvaziri S, Kooiker KB, Ellenberger M, Fajardo G, Zhao M, Vander Roest AS (2021). Altered Cardiac energetics and mitochondrial dysfunction in hypertrophic cardiomyopathy. Circulation.

[20] Xu YN, Cui XS, Sun SC, Lee SE, Li YH, Kwon JS (2011). Mitochondrial dysfunction influences apoptosis and autophagy in porcine parthenotes developing in vitro. J Reprod Dev.

[21] Gibson GE, Starkov A, Blass JP, Ratan RR, Beal MF (2010). Cause and consequence: mitochondrial dysfunction initiates and propagates neuronal dysfunction, neuronal death and behavioral abnormalities in age-associated neurodegenerative diseases. Biochim Biophys Acta.

[22] Peng C, Zhang Y, Lang X, Zhang Y (2023). Role of mitochondrial metabolic disorder and immune infiltration in diabetic cardiomyopathy: new insights from bioinformatics analysis. J Transl Med.

[23] Ritchie ME, Phipson B, Wu D, Hu Y, Law CW, Shi W (2015). Limma powers differential expression analyses for RNA-sequencing and microarray studies. Nucleic Acids Res.

[24] Gustavsson EK, Zhang D, Reynolds RH, Garcia-Ruiz S, Ryten M (2022). Ggtranscript: an R package for the visualization and interpretation of transcript isoforms using ggplot2. Bioinformatics.

[25] Yu G, Wang LG, Han Y, He QY (2012). clusterProfiler: an R package for comparing biological themes among gene clusters. Omics.

[26] Li Y, Lu F, Yin Y (2022). Applying logistic LASSO regression for the diagnosis of atypical Crohn’s disease. Sci Rep.

[27] Sachs MC. plotROC: A Tool for plotting ROC Curves. J Stat Softw. 2017;79. 10.18637/jss.v079.c02 PMC6347406 30686944

[28] Chen B, Khodadoust MS, Liu CL, Newman AM, Alizadeh AA (2018). Profiling Tumor infiltrating Immune cells with CIBERSORT. Methods Mol Biol.

[29] de Rojas I, Moreno-Grau S, Tesi N, Grenier-Boley B, Andrade V, Jansen IE (2021). Common variants in Alzheimer’s disease and risk stratification by polygenic risk scores. Nat Commun.

[30] Prokopidis K, Giannos P, Witard OC, Peckham D, Ispoglou T (2022). Aberrant mitochondrial homeostasis at the crossroad of musculoskeletal ageing and non-small cell lung cancer. PLoS ONE.

[31] Anvret A, Ran C, Westerlund M, Thelander AC, Sydow O, Lind C (2010). Possible involvement of a mitochondrial translation initiation factor 3 variant causing decreased mRNA levels in Parkinson’s disease. Parkinsons Dis.

[32] Satitsuksanoa P, Iwasaki S, Boersma J, Imam MB, Schneider SR, Chang I, van de Veen W, Akdis M (2023). B cells: the many facets of B cells in allergic diseases. J AllergyClin Immunol.

[33] Vogel RO, Janssen RJ, Ugalde C, Grovenstein M, Huijbens RJ, Visch HJ (2005). Human mitochondrial complex I assembly is mediated by NDUFAF1. Febs j.

[34] Tang Y, Yang X, Shu H, Yu Y, Pan S, Xu J (2021). Bioinformatic analysis identifies potential biomarkers and therapeutic targets of septic-shock-associated acute kidney injury. Hereditas.

[35] Wu Y, Chen H, Xuan N, Zhou L, Wu Y, Zhu C (2020). Induction of ferroptosis-like cell death of eosinophils exerts synergistic effects with glucocorticoids in allergic airway inflammation. Thorax.

[36] Hammad H, Lambrecht BN (2021). The basic immunology of asthma. Cell.

[37] Li J, Sha J, Sun L, Zhu D, Meng C (2021). Contribution of Regulatory T Cell Methylation Modifications to the pathogenesis of allergic Airway diseases. J Immunol Res.

[38] Burzyn D, Benoist C, Mathis D (2013). Regulatory T cells in nonlymphoid tissues. Nat Immunol.

[39] van der Veeken J, Gonzalez AJ, Cho H, Arvey A, Hemmers S, Leslie CS (2016). Memory of Inflammation in Regulatory T Cells. Cell.

[40] Feldman S, Kasjanski R, Poposki J, Hernandez D, Chen JN, Norton JE (2017). Chronic airway inflammation provides a unique environment for B cell activation and antibody production. Clin Exp Allergy.

[41] Lin C, Blessing AM, Pulliam TL, Shi Y, Wilkenfeld SR, Han JJ (2021). Inhibition of CAMKK2 impairs autophagy and castration-resistant prostate cancer via suppression of AMPK-ULK1 signaling. Oncogene.

[42] Lee J, Kim HS (2019). The role of Autophagy in Eosinophilic Airway Inflammation. Immune Netw.

[43] McAlinden KD, Deshpande DA, Ghavami S, Xenaki D, Sohal SS, Oliver BG (2019). Autophagy activation in Asthma airways remodeling. Am J Respir Cell Mol Biol.

[44] Martin LJ, Gupta J, Jyothula SS, Butsch Kovacic M, Biagini Myers JM, Patterson TL (2012). Functional variant in the autophagy-related 5 gene promotor is associated with childhood asthma. PLoS ONE.

[45] Qiu YN, Wang GH, Zhou F, Hao JJ, Tian L, Guan LF (2019). PM2.5 induces liver fibrosis via triggering ROS-mediated mitophagy. Ecotoxicol Environ Saf.

[46] Wu J, Dong F, Wang RA, Wang J, Zhao J, Yang M (2013). Central role of cellular senescence in TSLP-induced airway remodeling in asthma. PLoS ONE.

[47] Kang JY, Lee SY, Rhee CK, Kim SJ, Kwon SS, Kim YK (2013). Effect of aging on airway remodeling and muscarinic receptors in a murine acute asthma model. Clin Interv Aging.

[48] Wiley CD, Velarde MC, Lecot P, Liu S, Sarnoski EA, Freund A (2016). Mitochondrial dysfunction induces senescence with a distinct secretory phenotype. Cell Metab.

[49] Schuliga M, Pechkovsky DV, Read J, Waters DW, Blokland KEC, Reid AT (2018). Mitochondrial dysfunction contributes to the senescent phenotype of IPF lung fibroblasts. J Cell Mol Med.

[50] Alamro H, Bajic V, Macvanin MT, Isenovic ER, Gojobori T, Essack M, Gao X (2023). Type 2 diabetes Mellitus and its comorbidity, Alzheimer’s disease: identifying critical microRNA using machine learning. Front Endocrinol (Lausanne).

[51] Alamro H, Thafar MA, Albaradei S, Gojobori T, Essack M, Gao X (2023). Exploiting machine learning models to identify novel Alzheimer’s disease biomarkers and potential targets. Sci Rep.

[52] Zhang L, Hu S, Chen J, Ma S, Liu F, Liu C, Gao Y (2021). Comprehensive analysis of the MIR4435-2HG/miR-1-3p/MMP9/miR-29-3p/DUXAP8 ceRNA network axis in hepatocellular carcinoma. Discov Oncol.

