# Supplementary figures and images for: Identification of mitochondria-related biomarkers in childhood allergic asthma
Source: BMC Med Genomics. 2024 May 23;17:141. doi: 10.1186/s12920-024-01901-y (PMC11112767; doi:10.1186/s12920-024-01901-y)

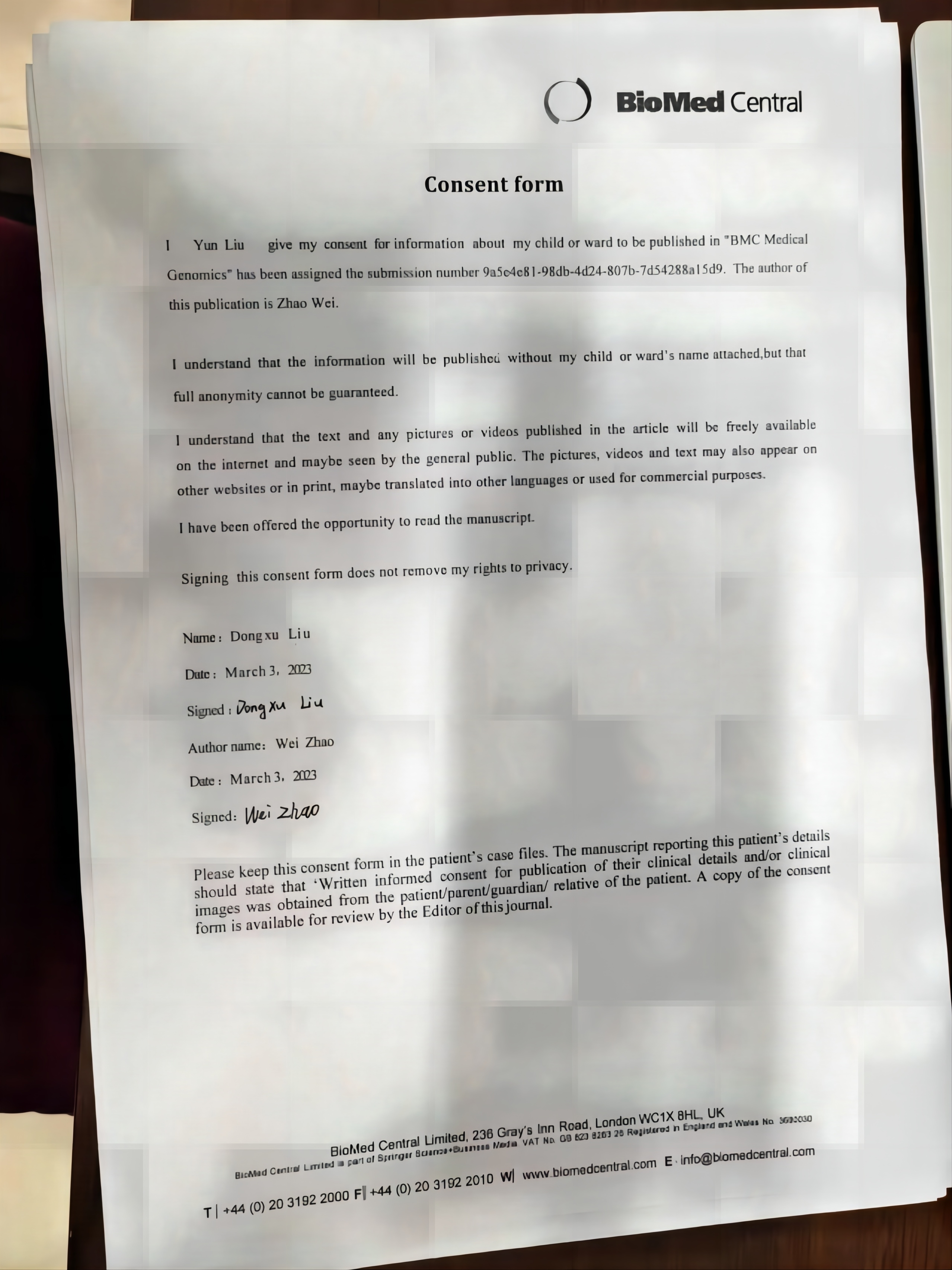

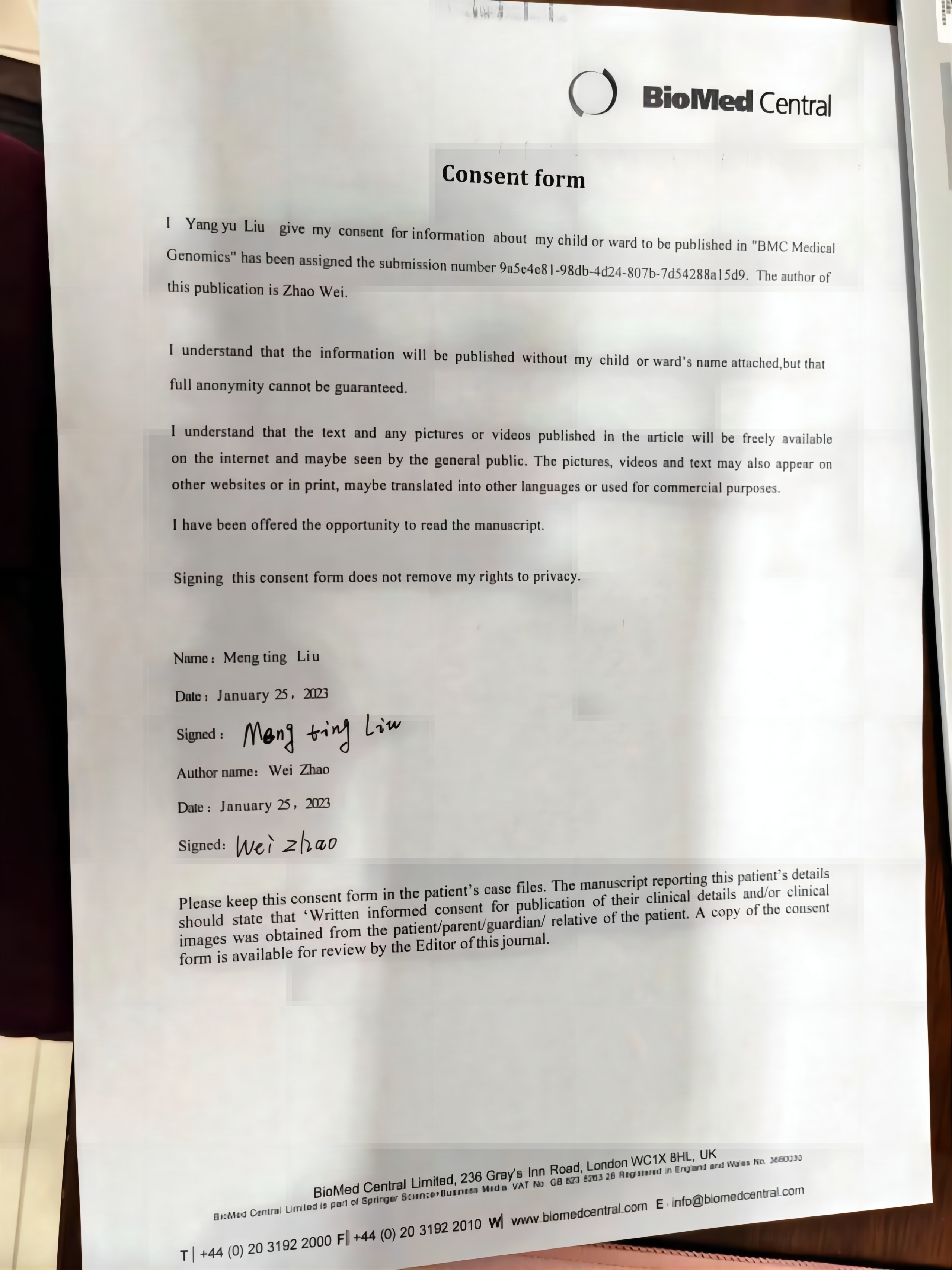

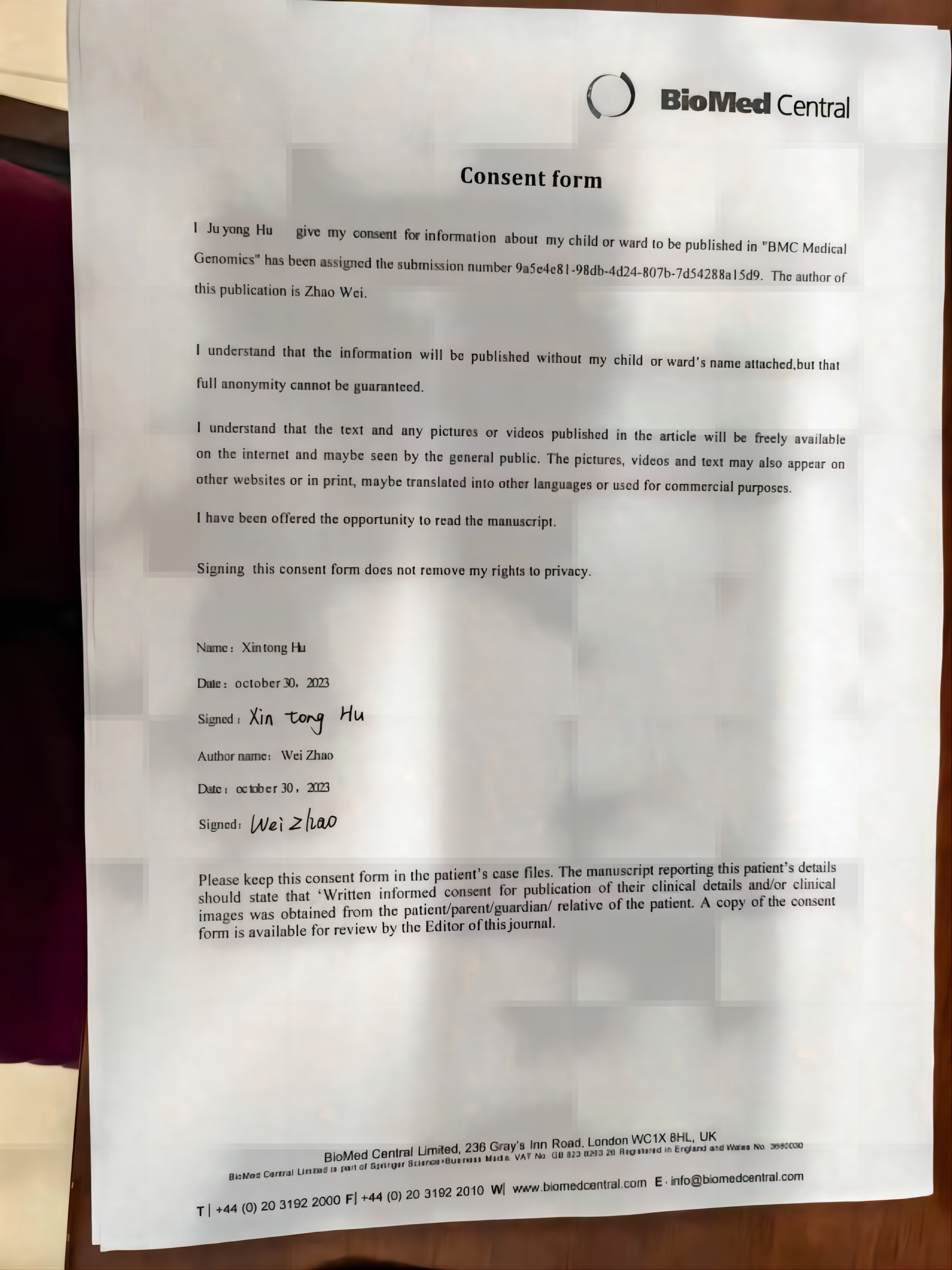

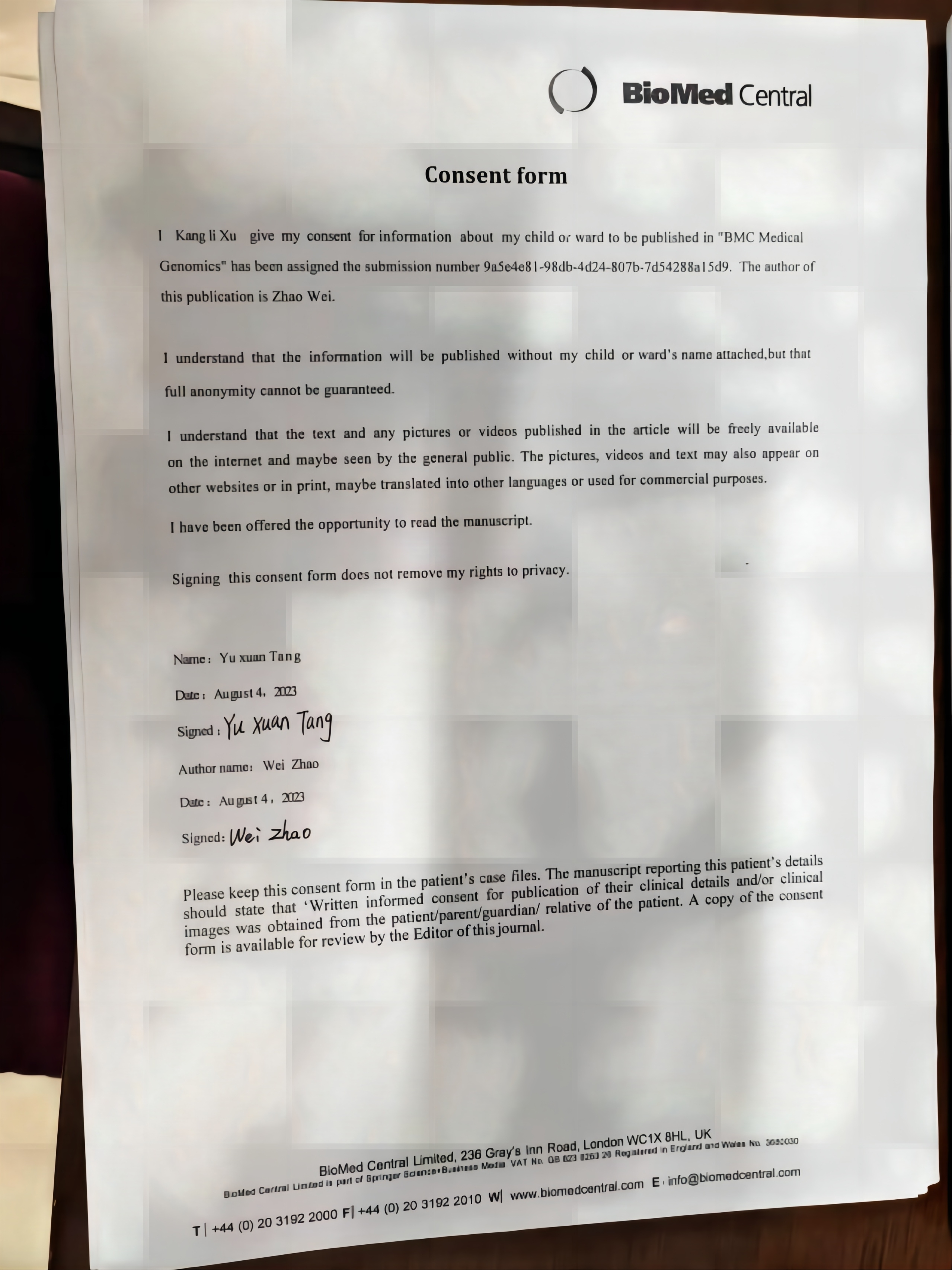

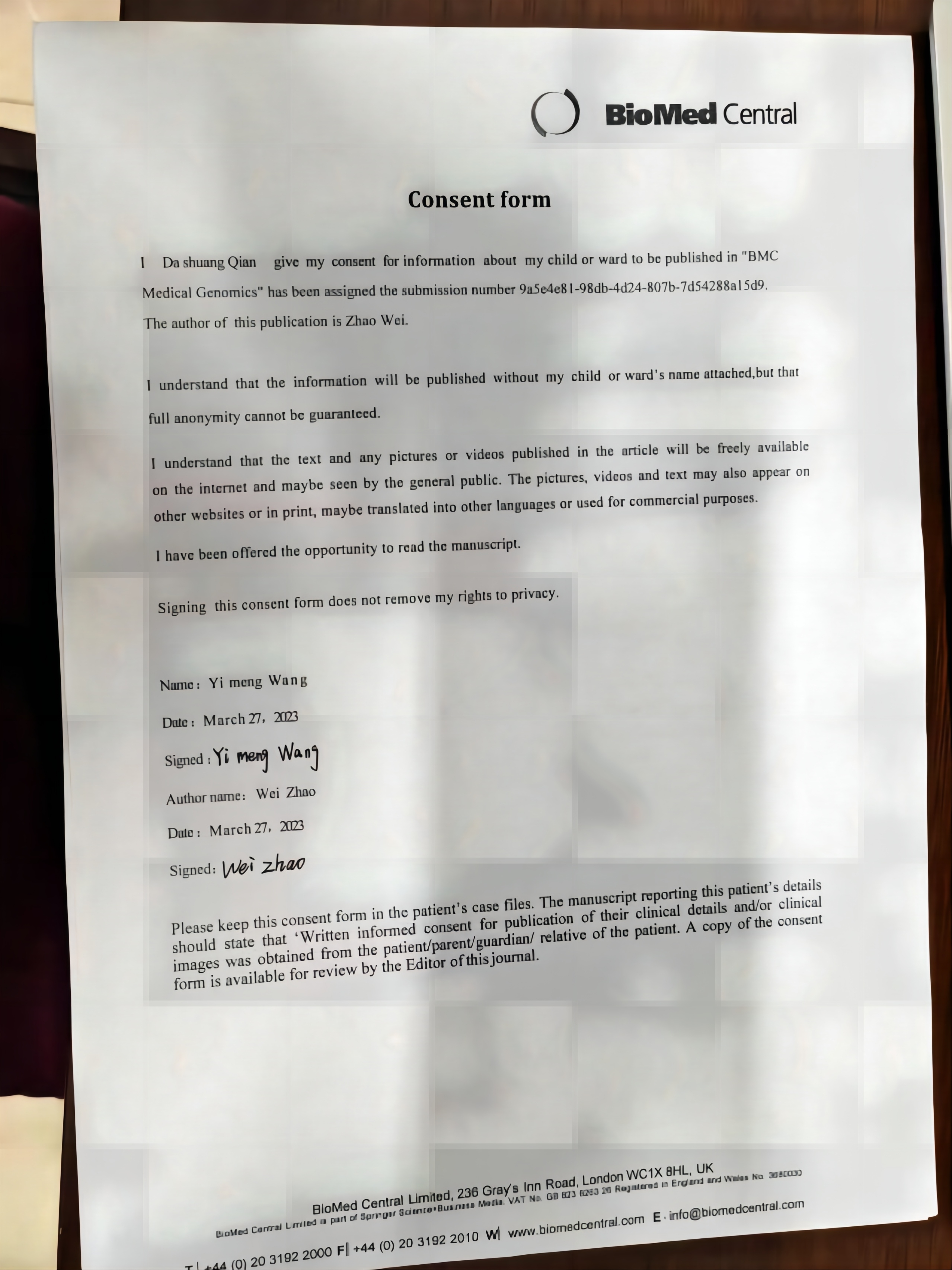

Supplement: Supplementary file 1 — Supplementary Material 1 [file 12920_2024_1901_MOESM1_ESM.docx]
